# Cervical Cancer Survival in Mali: A 21‐Year Retrospective Cohort Study of Trends, Determinants, and Outcomes at Gabriel Touré Teaching Hospital (2003–2024)

**DOI:** 10.1155/ogi/9966827

**Published:** 2026-09-17

**Authors:** Fatoumata Korika Tounkara, Abdoulaye Sissoko, Madi Traoré, Boulaye Diawara, Soumaila Sanogo, Ibrahima Téguété

**Affiliations:** ^1^ Faculty of Medicine and Odonto-Stomatology of Bamako at the University of Sciences Techniques and Technologies of Bamako (USTTB), Bamako, Mali; ^2^ Department of Gynecology and Obstetrics, Gabriel Touré Teaching Hospital, Bamako, Mali

**Keywords:** cervical cancer, Cox regression, Kaplan–Meier, low-income countries, Mali, prognostic factors, radiotherapy, sub-Saharan Africa, survival analysis

## Abstract

**Background and Aims:**

Cervical cancer (CC) remains a leading cause of cancer‐related mortality among women in sub‐Saharan Africa (SSA), particularly in Mali, where it is the most frequently diagnosed cancer and the leading cause of cancer‐related death among women. Despite recent efforts, including expanded screening initiatives and the introduction of radiotherapy, most women continue to be diagnosed at advanced stages. This study aimed to assess long‐term survival among women diagnosed with invasive CC at Gabriel Touré Teaching Hospital between 2003 and 2024 and to identify sociodemographic and clinical determinants of mortality.

**Methods:**

We conducted a retrospective cohort study including 3112 women with histologically confirmed invasive CC diagnosed between January 2003 and December 2024. Following data validation, 3083 women were included in survival analyses. Data were extracted from the hospital‐based cancer registry. Overall survival (OS) was defined as the time from diagnosis to death from any cause or last known contact and was estimated using the Kaplan–Meier method. Multivariable Cox proportional hazards models were used to identify predictors of mortality.

**Results:**

Median OS was 15.5 months (95% CI: 14.3–17.1). Survival rates were 82.4%, 65.8%, and 52.1% at 6, 12, and 24 months, respectively. Most patients were diagnosed at advanced stages, with 60.1% presenting with FIGO Stage IV disease, and 47.1% received palliative care only. Increased mortality was independently associated with anemia (AHR = 1.60; 95% CI: 1.45–1.76), menopausal status (AHR = 1.17; 95% CI: 1.04–1.32), lower educational attainment (no formal education: AHR = 1.47; 95% CI: 1.20–1.80; primary education: AHR = 1.57; 95% CI: 1.23–2.00), and advanced disease stage. Women diagnosed with FIGO Stage IV disease had the highest mortality risk (AHR = 4.95; 95% CI: 3.53–6.94). Compared with palliative care, radiotherapy alone, chemotherapy alone, radiotherapy plus chemotherapy, and multimodal treatment were associated with lower mortality.

**Conclusion:**

Among women receiving care at a major referral center in Mali, OS remained poor and was strongly associated with disease stage at diagnosis, anemia, and socioeconomic factors. Expanding early detection, improving access to specialized cancer care, and strengthening supportive services may contribute to improved survival outcomes.

**Summary**
• This study presents a 21‐year survival analysis of 3112 women with invasive cervical cancer in Mali, using data from the country’s largest referral hospital.
• Median overall survival was 15.5 months (95% CI: 14.3–17.1), with over 80% of patients diagnosed at an advanced FIGO stage (III or IV).
• Anemia, menopausal status, and low educational level were independently associated with increased mortality, while combined chemoradiotherapy significantly improved survival.
• Despite some recent progress, access to timely diagnosis and curative treatment remains highly unequal and centralized in urban areas.
• The study underscores the urgent need for early detection, treatment decentralization, and community‐based education to improve cervical cancer outcomes in low‐resource settings.

## 1. Introduction

Cervical cancer (CC) remains a major global public health challenge, disproportionately affecting women in low‐ and middle‐income countries (LMICs). In 2022, approximately 662,000 new cases and 349,000 deaths were reported worldwide, with nearly 90% occurring in LMICs, particularly in sub‐Saharan Africa (SSA) [1, 2]. These disparities reflect persistent inequities in access to prevention, early detection, and effective treatment.

CC is largely preventable through screening and human papillomavirus (HPV) vaccination, as it develops slowly from detectable precancerous lesions [3]. In high‐income countries, organized screening programs and HPV vaccination have substantially reduced incidence and mortality [4, 5]. In contrast, screening in SSA remains largely opportunistic and poorly integrated into routine health services, leading to late‐stage diagnosis and poor survival outcomes [5–8].

In Mali, CC is the most frequently diagnosed cancer and the leading cause of cancer‐related mortality among women [9]. Approximately 1934 new cases and 1406 deaths are reported annually, with high age‐standardized incidence and mortality rates [10]. National efforts have included visual inspection‐based screening, community initiatives such as the Weekend70 program, and the introduction of radiotherapy services in Bamako [11–15]. Despite these efforts, most women continue to present with advanced‐stage disease, limiting access to curative treatment.

Although previous studies have described incidence patterns and screening experiences in Mali [9, 11–15], important gaps remain regarding long‐term survival trends and their determinants. In particular, data are scarce on how survival has evolved over time in response to changes in screening and treatment availability.

This study therefore aimed to assess 21‐year overall survival (OS) trends (2003–2024) and identify key determinants of mortality among women with invasive CC treated at Gabriel Touré Teaching Hospital in Bamako.

## 2. Methods

### 2.1. Study Design and Setting

This retrospective cohort study was conducted at Gabriel Touré Teaching Hospital in Bamako, Mali, and included all women diagnosed with histologically confirmed invasive CC between January 1, 2003, and December 31, 2024. The hospital’s CC management unit, located within the Department of Gynecology and Obstetrics, plays a central role in the national response to CC and serves as a major referral center for diagnosis, staging, and initial management. Because the cohort was hospital‐based, it remains subject to referral and selection bias and should not be considered nationally representative.

**FIGURE 1:**
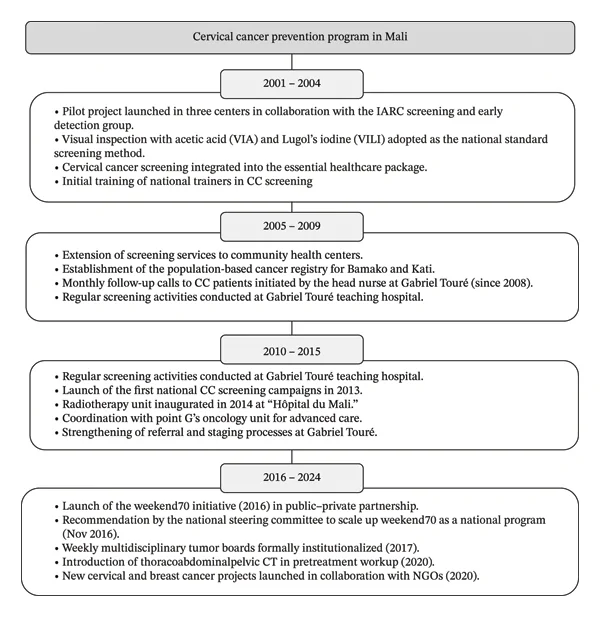
Key milestones in cervical cancer prevention and management in Mali (2001–2024). Progressive implementation of cervical cancer control efforts, from pilot VIA/VILI screening projects to nationwide initiatives such as Weekend70, establishment of radiotherapy services, and integration of multidisciplinary care. These milestones illustrate the evolution of Mali’s strategy to improve early detection, treatment access, and long‐term patient outcomes. Abbreviations: IARC, International Agency for Research on Cancer; VIA/VILI, visual inspection with acetic acid/visual inspection with Lugol’s iodine; CC, cervical cancer; NGO, nongovernmental organization.

According to national guidelines, CC screening is included in the essential healthcare package and should be routinely offered across all health system levels [16]. As illustrated in Figure 1, CC screening activities were introduced in 2001 and have expanded significantly over time. The introduction of radiotherapy in 2014 marked a major milestone in the management of advanced‐stage CC and contributed to improved prognosis [17].

### 2.2. CC Registry

A hospital‐based CC registry was established in 2003 within the Gynecologic and Breast Cancer Screening and Management Unit at Gabriel Touré Hospital. Data were abstracted from medical records using a standardized form by a team of four or five trained senior medical students each year, all nearing completion of their medical studies, under the supervision of the senior investigator and in accordance with IARC guidance. Every 2 weeks, the senior investigator reviewed the progress of data abstraction with the abstractors. During these review meetings, entries were examined for completeness, internal consistency, duplicate records, and implausible values; identified discrepancies were discussed, and appropriate corrections were made in the database on the basis of the available source documentation. When an inconsistency could not be resolved from the available records, the value was treated as missing or excluded from the relevant analysis. Formal duplicate abstraction and inter‐rater reliability statistics were not performed, which is a limitation of the retrospective registry.

### 2.3. Study Population

The registry was reviewed to identify all women diagnosed with histologically confirmed invasive CC between 2003 and 2024. Inclusion criteria were admission to the Department of Obstetrics and Gynecology with a new diagnosis of primary CC. Exclusion criteria included recurrent disease and malignancies originating outside the cervix.

### 2.4. Data Collection

Data were extracted using a standardized collection form. Sociodemographic variables included age, marital status, education level, occupation, region of origin, height, and weight. Gynecologic and obstetric history included menopausal status, parity, gravidity, and contraceptive use.

Clinical presentation variables included symptoms such as hydrorrhea, metrorrhagia, pelvic mass, urinary or rectal signs, and symptom duration. Tumor characteristics comprised FIGO stage, histological type, grade of differentiation, and presence of complications or metastases (urinary tract infection, hydronephrosis, vesicovaginal fistula, cerebral/liver/lung metastases, etc.). Ancillary investigations included complete blood count, creatinine levels, Rhesus typing, pelvic ultrasound, CT scan, and intravenous urography. Treatment‐related variables included surgery, chemotherapy, radiotherapy, and associated complications.

### 2.5. CC Management

CC treatment at Gabriel Touré follows institutional protocols aligned with international oncology standards, adapted to local resource constraints. Treatment is determined based on FIGO stage. Early‐stage disease (FIGO I‐IIA) is typically managed with radical surgery, often followed by adjuvant chemotherapy and/or radiotherapy. Advanced‐stage disease (FIGO IIB‐IV) is treated primarily with concurrent chemoradiotherapy.

Due to limited infrastructure, Mali has only one radiotherapy center. Treatment delays of 3–9 months are common, necessitating the use of alternative regimens (surgery or chemotherapy alone) when radiotherapy is not immediately available.

Standardized preradiotherapy assessments include clinical examination, complete blood count, renal function, chest X‐ray, and urography. Since 2020, thoracoabdominalpelvic CT has been integrated into the pretreatment workup. External beam radiotherapy is delivered in two phases totaling 70 Gy. Phase I (46 Gy over 23 sessions) targets the uterus and pelvic lymph nodes using four‐field techniques. Phase II (24 Gy over 12 sessions) targets the residual tumor volume. A tumor‐directed boost of 30 Gy (3 Gy × 10 fractions) is administered. Concurrent weekly cisplatin (40 mg/m^2^) is used as a radiosensitizer.

Treatment categories were based on treatment plans documented in the medical records and cancer registry. Information on treatment completion, adherence, or treatment interruptions was not consistently available throughout the study period and therefore could not be assessed.

### 2.6. Palliative Care

Palliative care constitutes a critical component of CC management in Mali, especially for women with advanced or nonoperable disease. However, access to formal palliative care remains limited and largely confined to tertiary centers in Bamako.

At Gabriel Touré, palliative care is delivered by gynecologists and nurses with limited specialized infrastructure. Management focuses on symptomatic relief of pain, bleeding, and urinary/rectal complications using oral morphine, antibiotics, catheterization, wound care, and other supportive measures. Access to morphine is inconsistent due to procurement and regulatory barriers. Psychosocial and home‐based palliative services are underdeveloped. Despite the adoption of a national palliative care policy in 2017, its operationalization remains limited due to funding and training gaps. These contextual factors are important for interpreting survival outcomes in this study.

### 2.7. Posttreatment Follow‐Up

Patients completing curative treatment were scheduled for follow‐up visits at 3, 6, 12, 18, and 24 months, then annually. Follow‐up evaluations included clinical assessment, pelvic examination, and investigations as needed (ultrasound, CT, X‐ray, MRI, or biopsy). To minimize loss to follow‐up, the Chief Nursing Officer conducted monthly phone calls. If the patient could not be reached, contact was made with family members to ascertain vital and clinical status.

### 2.8. Outcome Definition

The primary outcome was OS, defined as the time (in months) from histopathological diagnosis to death from any cause or last known contact (through a clinic visit, telephone call, or notification). Vital status was obtained from medical records or verified through telephone contact with the patient or family members. Women alive at the last known contact were censored at that date. Cancer‐specific survival could not be assessed because reliable cause‐of‐death information was not consistently available. This outcome reflects real‐world survivorship in a resource‐limited setting, capturing the cumulative impact of clinical stage at diagnosis, treatment availability, and systemic care constraints, including access to palliative services.

### 2.9. Statistical Analysis

All statistical analyses were conducted using SAS Version 9.4 (SAS Institute Inc., Cary, NC, USA). Categorical variables were summarized using frequencies and percentages, while continuous variables were described using means with standard deviations (±SDs) or medians with interquartile ranges (IQR), as appropriate based on the distribution.

OS was defined as the time (in months) from histopathological diagnosis to the date of death or last follow‐up. Patients who were alive at last contact were right‐censored at that date. The Kaplan–Meier method was used to estimate survival probabilities, and survival curves were compared between groups using the log‐rank test. Median survival time and cumulative survival at 6, 12, and 24 months were reported.

To identify factors independently associated with mortality, a multivariable Cox proportional hazards regression model was constructed to estimate adjusted hazard ratios (AHRs) and their 95% confidence intervals (95% CI). The proportional hazards assumption was tested using Schoenfeld residuals and visual inspection of log–log survival plots. Candidate variables were selected on the basis of clinical relevance and prior evidence and included age, FIGO stage, histologic type, anemia at admission, treatment modalities (surgery, chemotherapy, and radiotherapy), metastases, and delay between symptom onset and admission. HIV status was not included in the Cox regression analysis because women with a confirmed negative test could not be reliably distinguished from women who had not been tested. Variables with *p* values < 0.20 in univariate analysis were initially retained. Manual backward elimination was then applied; covariates were sequentially excluded when *p* > 0.05 and their removal altered the coefficients of remaining variables by less than 10%.

Additionally, stratified analyses were conducted to examine survival differences across distinct calendar periods (2003–2009, 2010–2015, and 2016–2024), corresponding to major milestones in national CC control (screening scale‐up, radiotherapy introduction, and the Weekend70 initiative). All tests were two‐sided, and a *p*‐value < 0.05 was considered statistically significant.

### 2.10. Follow‐Up and Sensitivity Analysis

Vital status was ascertained through review of medical records, scheduled clinical follow‐up visits, and active telephone contact with patients or their relatives when necessary. Prior to survival analyses, records with implausible negative survival times resulting from inconsistent date information were identified during data validation. Twenty‐nine records (0.9%) were excluded because reliable correction was not possible.

OS was defined as the time from diagnosis to death from any cause or last known contact and was expressed in months.

To evaluate the potential impact of loss to follow‐up on survival estimates, a sensitivity analysis was performed under a worst‐case scenario in which all patients lost to follow‐up were assumed to have died at their last recorded contact. Survival estimates obtained from this analysis were compared with those from the primary Kaplan–Meier analysis.

### 2.11. Ethical Consideration

This study was approved by the Ethics Committee of the Faculty of Medicine and Pharmacy, University of Bamako (Approval No. 2020/233/CE/FMOS/FAPH). The study was conducted using secondary data extracted from the hospital‐based cancer registry and medical records. Because of the retrospective nature of the study and the use of routinely collected anonymized data, individual informed consent for participation in this research was waived by the Ethics Committee. All data were deidentified before analysis to ensure patient confidentiality and privacy.

## 3. Results

### 3.1. Sociodemographic Characteristics

Among the 3213 women diagnosed with CC, 29 in situ carcinomas and 72 cases with unknown histological type were excluded, leaving 3112 patients with confirmed invasive CC for analysis (Table 1).

**TABLE 1:** Sociodemographic and obstetric characteristics of patients with cervical cancer at Gabriel Touré Teaching Hospital, Bamako.

Characteristics	Number or mean (% or SD)
Sociodemographic characteristics	
Age at diagnosis in years, mean (±SD)	51.27 ± 13.05
Age interval at diagnosis in years, *n* (%)	
< 30	98 (3.2)
30–39	480 (15.4)
40–49	778 (25.0)
50–59	826 (26.5)
≥ 60	930 (29.9)
Education level^∗^	
Uneducated	2482 (79.8)
Primary	300 (9.6)
Secondary or higher	250 (8.0)
Unknown	80 (2.6)
Occupation	
Housewife	2665 (85.6)
Businesswomen	191 (6.1)
Others^∗∗^	254 (8.2)
Marital status	
Married	2460 (79.1)
Single^$^	652 (20.9)
Types of matrimonial regime	
Monogamy	1010 (32.5)
Polygamy	1877 (60.3)
Single	225 (7.2)
Provenance of patients	
Kayes	239 (7.7)
Koulikoro	337 (10.8)
Sikasso	216 (6.9)
Segou	144 (4.6)
Mopti	93 (3.0)
Tombouctou	21 (0.7)
Gao	8 (0.3)
Kidal	2 (0.1)
Bamako	1955 (62.2)
Others	117 (3.7)
Body mass index (Kg/m^2^)	
< 30	2815 (90.5)
≥ 30	158 (5.1)
Unknown	139 (4.4)
Gynecological and obstetrical characteristics	
Uses of modern contraceptive methods	
Yes	527 (17.0)
No	2575 (83.0)
Number of parities	
Primiparous	214 (6.9)
Pauciparous	301 (9.7)
Multiparous	934 (30.0)
Grand multiparous	1662 (53.4)
Menopausal status	
Menopausal	1825 (58.6)
Premenopausal	1287 (41.4)

*Note:* Missing values are not mutually exclusive; SCC, squamous cell.

Abbreviations: IQR, interquartile range; SD, standard deviation.

^∗^Measured as the highest level of education attained.

^∗∗^Others, civil servant, or others.

^$^Divorced, widowed, or never‐married women.

Most women were from Bamako (62.2%), followed by Koulikoro (10.8%), Kayes (7.7%), Sikasso (6.9%), Ségou (4.6%), Mopti (3.0%), Tombouctou (0.7%), Gao (0.3%), and Kidal (0.1%). The mean age at diagnosis was 51.3 years, with 56.4% aged 50 or older. The majority were uneducated (79.8%), housewives (85.6%), and married (79.1%), including 60.3% in polygamous unions. Only 17.0% had ever used modern contraceptives. More than half (53.4%) were grand multiparous, and 58.6% were postmenopausal. Most women (90.5%) had a BMI < 30 kg/m^2^, whereas 9.5% had a BMI ≥ 30 kg/m^2^.

### 3.2. Clinical Presentation and Treatment

Among the 3112 women with invasive CC, metrorrhagia was the most common symptom (65.4%), followed by hydrorrhea (31.6%), urinary symptoms (12.1%), and rectal symptoms (9.0%) (Table 2). Anemia was observed in 48.4%. HIV positivity was recorded for 4.2% of the overall cohort. Pelvic mass was recorded in 2.4%. Squamous cell carcinoma predominated (91.2%), with most patients diagnosed at advanced stages; 60.1% had FIGO Stage IV disease, and 11.4% had distant metastases. Hydronephrosis occurred in 13.9% of cases.

**TABLE 2:** Clinical presentation, histopathological features, and treatment modalities among patients with cervical cancer at Gabriel Touré Teaching Hospital, Bamako.

Characteristics	Number or mean (% or SD)
Clinical sign and reason for consultation at admission	
HIV status	
Positive	130 (4.2)
Negative	2965 (95.8)
Anemia at admission	
Hemoglobin level < 11 g/dL	1492 (48.4)
Hemoglobin level ≥ 11 g/dL	1590 (51.6)
Hydrorrhea sign at admission	
Yes	982 (31.6)
No	2130 (68.4)
Pelvic mass sign at admission	
Yes	76 (2.4)
No	3036 (97.6)
Metrorrhagia sign at admission	
Yes	2035 (65.4)
No	1077 (34.6)
Rectal symptoms at admission	
Yes	280 (9.0)
No	2832 (91.0)
Urinary symptoms at admission	
Yes	375 (12.1)
No	2737 (87.9)
Clinical complications	
Histology type	
SCC	2733 (91.2)
Adenocarcinoma	218 (7.3)
Others^a^	45 (1.5)
Tumor staging	
In situ Stage 0	
Stage I	207 (6.7)
Stage II	408 (13.1)
Stage III	628 (20.1)
Stage IV	1869 (60.1)
Urinary infection	
Yes	32 (1.0)
No	3042 (99.0)
Hydronephrosis	
Yes	426 (13.9)
No	2646 (86.1)
Metastases	
Yes	349 (11.4)
No	2705 (88.6
Cerebral metastases	
Yes	10 (0.3)
No	3029 (99.7)
Hepatic metastases	
Yes	103 (3.4)
No	2968 (96.6)
Pulmonary metastases	
Yes	130 (4.2)
No	2938 (95.8)
Rectal metastases	
Yes	161 (5.3)
No	2907 (94.7)
Treatment options	
Surgery only	215 (6.9)
Radiotherapy only	281 (9.0)
Chemotherapy only	177 (5.7)
Radiotherapy + chemotherapy	873 (28.0)
Multimodal	101 (3.3)
Palliative care	1465 (47.1)
Follow‐up for all patients (months), median (IQR), from diagnosis, time periods	13 (7–24)
2003–2009	707 (22.7)
2010–2015	1084 (34.8)
2016–2024	1321 (42.5)

*Note:* Missing values are not mutually exclusive.

Abbreviations: HIV, human immunodeficiency virus; IQR, interquartile range; SCC, squamous cell carcinoma; SD, standard deviation.

^a^Others, melanoma, neoplasm, sarcoma, and lymphoma.

### 3.3. Kaplan–Meier Survival Analysis

**FIGURE 2:**
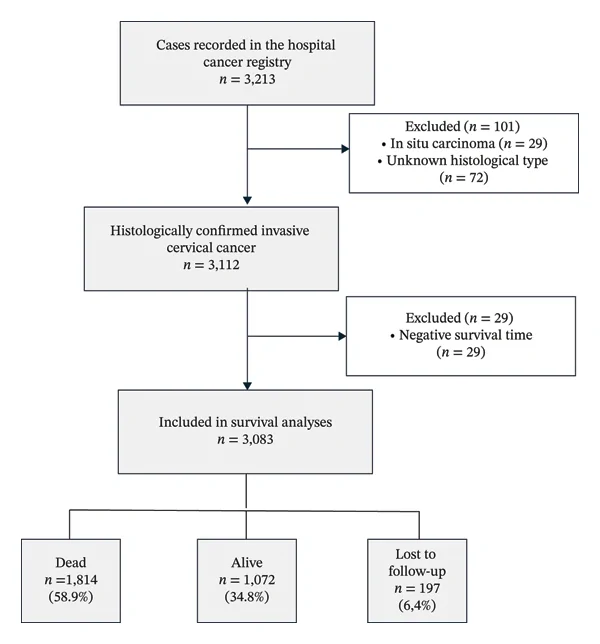
Flowchart of patient selection and inclusion in survival analyses at Gabriel Touré Teaching Hospital, Mali (2003–2024).

During data validation, 29 records (0.9%) with implausible negative survival times were excluded from survival analyses, leaving 3083 women for Kaplan–Meier and Cox regression analyses (Figure 2).

Follow‐up status was successfully ascertained for most patients. Among the 3083 women included in the survival analyses, 1814 (58.8%) were confirmed dead during follow‐up, 1072 (34.8%) were alive at the last known contact, and 197 (6.4%) were lost to follow‐up.

**FIGURE 3:**
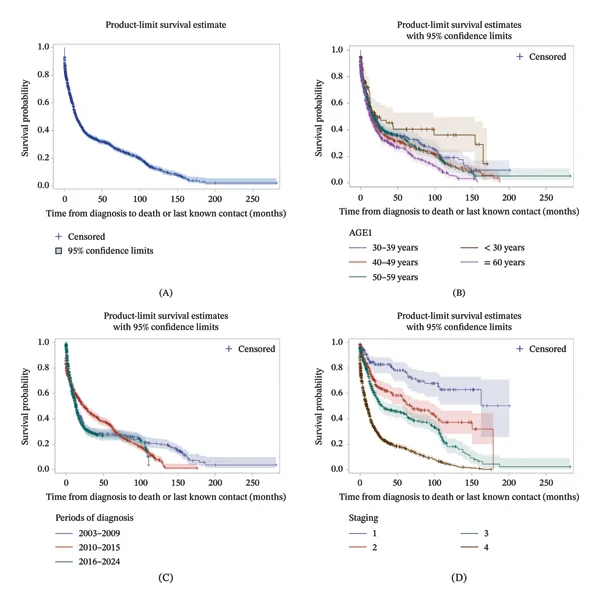
Kaplan–Meier overall survival curves among women diagnosed with invasive cervical cancer at Gabriel Touré Teaching Hospital, Mali (2003–2024). (A) Kaplan–Meier overall survival curve among women with invasive cervical cancer. Shaded areas represent 95% confidence intervals. (B) Survival curves stratified by age group. (C) Survival curves according to period of diagnosis. (D) Survival curves by FIGO stage at diagnosis.

Kaplan–Meier analysis was performed among 3083 women included in the final survival analyses. Median OS was 15.5 months (95% CI: 14.3–17.1) (Figure 3A‐D). Survival probabilities declined progressively over time, with the steepest decrease observed during the first year following diagnosis.

Survival differed significantly according to age group, with women younger than 40 years exhibiting higher survival probabilities than women aged 60 years and older (log‐rank *p* < 0.001). Survival also varied across calendar periods of diagnosis, with women diagnosed between 2016 and 2024 demonstrating significantly better survival than those diagnosed in earlier periods (log‐rank *p* < 0.01).

As expected, survival decreased with advancing FIGO stage (log‐rank *p* < 0.001). Twelve‐month survival exceeded 70% among women diagnosed at Stage I but was below 20% among those diagnosed at Stage IV.

In the sensitivity analysis assuming that all patients lost to follow‐up had died at their last recorded contact, median OS was 14.1 months (95% CI: 13.1–15.3), and the overall interpretation of the findings remained unchanged.

### 3.4. Factors Associated With Survival

Multivariable Cox regression identified several independent predictors of mortality (Table 3). Compared with women who had secondary or higher education, mortality risk was significantly higher among women with no formal education (AHR = 1.47; 95% CI: 1.20–1.80) and those with primary education (AHR = 1.57; 95% CI: 1.23–2.00). Menopausal status (AHR = 1.17; 95% CI: 1.04–1.32) and anemia at diagnosis (AHR = 1.60; 95% CI: 1.45–1.76) were also independently associated with increased mortality.

**TABLE 3:** Univariate and multivariate analysis using the Cox model of factors affecting cervical cancer survival at Gabriel Toure Teaching Hospital, 2003 to 2021.

Variables	Crude HR [CI 95%]	*p*‐value	AHR [CI 95%]	*p*‐value
Diagnosis, time periods		0.572		0.005
2003–2009			1.00	
2010–2015	0.98 [0.88–1.11]		0.87 [0.77–0.99]	
2016–2024	1.04 [0.92–1.18]		1.17 [1.01–1.36]	
Age interval at diagnosis in years, *n* (%)		< 0.0001		0.176
< 30	1.00		1.00	
30–39	1.44 [1.04–1.98]		1.39 [1.01–1.92]	
40–49	1.58 [1.16–2.16]		1.45 [1.06–1.99]	
50–59	1.59 [1.16–2.17]		1.31 [0.95–1.82]	
≥ 60	1.91 [1.40–2.60]		1.37 [0.99–1.88]	
Marital status		0.001		0.857
Married	0.84 [0.75–0.93]		0.99 [0.88–1.11]	
Single^∗^	1.00		1.00	
Education level^∗∗^		< 0.0001		0.000
Uneducated	1.99 [1.63–2.43]		1.47 [1.20–1.80]	
Primary	1.92 [1.51–2.45]		1.57 [1.23–2.00]	
Secondary or higher	1.00		1.00	
Menopausal status		< 0.0001		0.001
Menopausal	1.26 [1.14–1.38]		1.17 [1.04–1.32]	
Premenopausal	1.00		1.00	
Anemia at diagnosis		< 0.0001		< 0.0001
Hemoglobin level < 11 g/dL	1.75 [1.60–1.92]		1.60 [1.45–1.76]	
Hemoglobin level ≥ 11 g/dL	1.00		1.00	
Tumor staging		< 0.0001		< 0.0001
Stage I	1.00		1.00	
Stage II	2.12 [1.49–3.02]		1.76 [1.22–2.56]	
Stage III	3.17 [2.29–4.40]		2.48 [1.75–3.52]	
Stage IV	7.11 [5.20–9.72]		4.95 [3.53–6.94]	
CC treatment option		< 0.0001		< 0.0001
Surgery only	0.49 [0.41–0.60]		0.87 [0.70–1.08]	
Radiotherapy only	0.64 [0.54–0.76]		0.75 [0.63–0.90]	
Chemotherapy only	0.42 [0.32–0.55]		0.31 [0.23–0.42]	
Radiotherapy + chemotherapy	0.55 [0.49–0.62]		0.55 [0.48–0.62]	
Multimodal	0.25 [0.18–0.37]		0.54 [0.37–0.80]	
Palliative care	1.00		1.00	

Abbreviations: AHR, adjusted hazard ratio; CC, cervical cancer; CI, confidence interval; HR, hazard ratio.

^∗^Divorced, widowed, or never‐married women.

^∗∗^Measured as the highest level of education attained. Multivariate model was adjusted for all variables presented.

Tumor stage had the strongest association with survival. Compared with women diagnosed at FIGO Stage I, mortality risk increased progressively for Stage II (AHR = 1.76; 95% CI: 1.22–2.56), Stage III (AHR = 2.48; 95% CI: 1.75–3.52), and Stage IV disease (AHR = 4.95; 95% CI: 3.53–6.94).

Compared with palliative care, receipt of radiotherapy alone (AHR = 0.75; 95% CI: 0.63–0.90), chemotherapy alone (AHR = 0.31; 95% CI: 0.23–0.42), radiotherapy plus chemotherapy (AHR = 0.55; 95% CI: 0.48–0.62), and multimodal treatment (AHR = 0.54; 95% CI: 0.37–0.80) were associated with lower mortality.

## 4. Discussion

This 21‐year retrospective cohort study conducted at Gabriel Touré University Hospital in Bamako provides a comprehensive analysis of survival outcomes and prognostic factors among women diagnosed with invasive CC in Mali. The results reveal persistently poor survival, with a median OS of 15.5 months (95% CI: 14.3–17.1). At the time of diagnosis, many patients presented with advanced clinical signs, including metrorrhagia, foul‐smelling vaginal discharge, and anemia, and more than half were diagnosed at FIGO Stage IV. Additionally, 10% of patients had distant metastases at presentation, reflecting a striking delay in diagnosis and care. Multivariable analysis identified several significant predictors of overall mortality. Menopausal status, the presence of anemia, advanced disease stage, and the type of treatment received were independently associated with survival.

These results align with trends observed across SSA. A systematic review by Emagneneh et al. [18], synthesizing data from 23 African cohorts, estimated a 5‐year OS rate of just 35%, with wide variability, from 7% in Kenya to 60% in South Africa. Contributing factors include late‐stage diagnosis, limited access to surgery, radiotherapy, and chemotherapy, treatment abandonment, and systemic vulnerabilities, such as poverty, HIV coinfection, and inadequate health infrastructure [18, 19].

Geographic disparities were also notable in our cohort. Although Gabriel Touré is a major national referral center for CC care, 62.2% of patients in our cohort resided in Bamako, with less than 40% coming from rural regions. This reflects both the centralization of oncology services and broader inequities in health access. Studies conducted in Mali have reported frequent disruptions in CC screening at the primary care level due to shortages of acetic acid, Lugol’s iodine, and low staff motivation [14]. However, the implementation of the Weekend70 initiative, which offered free screening on weekends with community mobilization, markedly improved coverage in Bamako [15]. In our cohort, women diagnosed after 2015, particularly during or following the screening phases, showed modest improvements in survival, supporting the potential of targeted outreach programs to facilitate earlier diagnosis.

Sociodemographic characteristics also significantly influenced survival. Educational level emerged as a key predictor: Women with only a primary education had a 57% higher risk of death, likely due to limited health literacy, delayed symptom recognition, and difficulty navigating referral systems. This aligns with findings from Ghana, where authors identified low education and gender norms as major barriers to timely care. Menopausal status was also associated with worse outcomes [20]. Biologically, hormonal decline may reduce mucosal immunity and allow persistent HPV infection, while culturally, older women may dismiss gynecologic symptoms or delay care‐seeking due to stigma or perceived low risk [21].

From a clinical standpoint, advanced FIGO stage at diagnosis remained the most powerful predictor of mortality. As observed in studies from Morocco and Uganda, late‐stage presentation severely limits curative options and diminishes progression‐free survival [18, 21]. Anemia observed in nearly half of our cohort was another strong negative prognostic factor. In addition to reflecting advanced disease or nutritional deficiency, anemia reduces tumor oxygenation, impairing the effectiveness of radiotherapy. Evidence suggests that correcting anemia prior to treatment improves radiotherapy response and survival [18].

Despite adherence to treatment protocols aligned with international standards, access to optimal therapy remains a major challenge. Although combined chemoradiotherapy was associated with improved survival (AHR = 0.55; 95% CI: 0.48–0.62), only a small proportion of patients received it. Radiotherapy is available only in Bamako, with waiting times of 3–9 months, leading to reliance on incomplete or alternative regimens (such as surgery or chemotherapy alone) in many cases. This limitation must be recognized as a central driver of poor outcomes.

Palliative care, although essential for patients with inoperable or advanced disease, remains limited. At Gabriel Touré, palliative care is provided by gynecologists and nurses with minimal infrastructure or access to essential medications like morphine. Although Mali adopted a national palliative care policy in 2017, its implementation remains incomplete due to funding and training constraints. This gap further contributes to patient suffering and unmet care needs.

Patient follow‐up was another major challenge. While structured follow‐up visits were scheduled and supported by proactive phone tracking, some degree of loss to follow‐up, especially in later years, may have led to a modest underestimation of survival. Additionally, the absence of data on HPV genotypes, time to treatment initiation, quality of life, and patient‐reported outcomes limits a more nuanced understanding of prognosis.

According to SURVCAN‐3, the 3‐year standardized survival for CC in Bamako is only 12.4%, among the lowest in SSA [22]. This finding reinforces the urgent need for national investments in early detection, equitable access to treatment, palliative care services, and cancer registry strengthening to improve CC outcomes in Mali.

Loss to follow‐up represented a potential source of bias but affected only 6.4% of the cohort. To assess its impact, we conducted a sensitivity analysis under an extreme‐case scenario in which all patients lost to follow‐up were assumed to have died at their last recorded contact. The resulting survival estimates differed only modestly from those of the primary analysis (median OS: 15.5 vs. 14.1 months), suggesting that the overall conclusions were robust to potential outcome misclassification. Nevertheless, because cancer care remains highly centralized in Bamako and no national mortality registry exists, some deaths occurring outside the treating institution may not have been captured. In addition, a small number of patients may have continued treatment outside the country and therefore ceased follow‐up at our institution.

### 4.1. Limitations

First, this was a retrospective hospital‐based cohort rather than a population‐based study. Nevertheless, CC diagnosis and treatment services were largely centralized in Bamako during the study period, and Gabriel Touré Teaching Hospital functioned as a major national referral center. Because many women from regional areas temporarily relocated to Bamako during treatment, recorded residence may not accurately reflect geographic origin. Therefore, although the findings should not be interpreted as population‐based estimates, they provide important evidence regarding survival patterns among women who accessed specialized CC care in Mali.

Second, although active follow‐up was conducted through scheduled visits and telephone contact with patients or their relatives, outcome ascertainment partly relied on indirect reporting. Consequently, some degree of outcome misclassification may have occurred. However, only 197 women (6.4%) were lost to follow‐up among the 3083 included in the survival analyses. Furthermore, a sensitivity analysis assuming that all patients lost to follow‐up had died at their last recorded contact yielded similar survival estimates, suggesting that the principal findings were robust to potential follow‐up bias.

Third, OS was based on all‐cause mortality rather than CC‐specific mortality because reliable cause‐of‐death information was not consistently available. Consequently, mortality attributable specifically to CC may have been overestimated, particularly among older patients with competing health conditions.

Fourth, treatment categories were based on assigned treatment plans rather than confirmed treatment completion. Information on treatment adherence, interruptions, and completion was not consistently available, which may have introduced some degree of treatment misclassification and influenced treatment‐survival associations. In addition, treatment allocation was determined by clinical presentation, disease stage, and treatment availability rather than random assignment. Therefore, treatment‐related findings should be interpreted as associations and not as evidence of causal treatment effects.

Finally, several potentially important variables, including HIV status, detailed tumor characteristics, HPV genotype, treatment delays, and patient‐reported outcomes, were incompletely recorded or unavailable in the registry. HIV status was therefore reported descriptively but excluded from the multivariable Cox model. In addition, formal inter‐rater reliability among the annual teams of abstractors was not assessed. Although the models adjusted for available prognostic factors, residual confounding and measurement error cannot be excluded.

### 4.2. Study Implications

This study highlights several priorities for improving CC outcomes in Mali and similar low‐resource settings. The poor survival observed in this cohort, together with the high proportion of women diagnosed with advanced‐stage disease, underscores the importance of expanding early detection through screening programs, primary healthcare services, community outreach, and public awareness initiatives. The strong associations observed between anemia, educational attainment, disease stage, and mortality suggest that both clinical and social determinants influence survival outcomes and should be addressed through comprehensive cancer control strategies.

The findings also highlight persistent challenges related to access to specialized cancer care. During most of the study period, radiotherapy services were available only in Bamako, creating important geographic and financial barriers for women living outside the capital. Efforts to reduce treatment delays, improve referral pathways, strengthen supportive care, and expand access to comprehensive oncology services may contribute to better outcomes. Finally, continued investment in cancer registration systems and long‐term follow‐up mechanisms is essential to improve surveillance, evaluate interventions, and support evidence‐based cancer control policies.

## 5. Conclusion

This 21‐year hospital‐based cohort study provides important evidence on CC survival among women receiving care at a major referral center in Mali. OS remained poor and was strongly associated with advanced stage at diagnosis, anemia, menopausal status, and lower educational attainment. Women diagnosed with Stage IV disease experienced the highest mortality risk.

These findings emphasize the importance of timely diagnosis, timely access to specialized treatment, and strengthened supportive care services. Expanding screening activities, reducing barriers to cancer care, and improving cancer surveillance systems may contribute to improved survival outcomes. Continued efforts to strengthen CC prevention, diagnosis, treatment, and follow‐up services will be essential to reduce the burden of CC in Mali.

**Nomenclature**
AHR: Adjusted hazard ratio
BMI: Body mass index
CC: Cervical cancer
CI: Confidence interval
CT: Computed tomography
FIGO: Fédération Internationale de Gynécologie et d’Obstétrique (International Federation of Gynecology and Obstetrics)
Gy: Gray (unit of radiation dose)
HIV: Human immunodeficiency virus
HPV: Human papillomavirus
IARC: International Agency for Research on Cancer
IQR: Interquartile range
LMICs: Low‐ and middle‐income countries
MRI: Magnetic resonance imaging
MSF: Médecins Sans Frontières
OS: Overall survival
SSA: Sub‐Saharan Africa
SURVCAN: Cancer survival in Africa, Asia, the Caribbean and Central America project
VIA: Visual inspection with acetic acid
VILI: Visual inspection with Lugol’s iodine
WHO: World Health Organization

## Author Contributions

Fatoumata Korika Tounkara led the drafting of the manuscript and performed data cleaning, analysis, and interpretation. She also supervised data collection and contributed significantly to the revision of the database. Madi Traoré and Boulaye Diawara coordinated cervical cancer screening at Gabriel Touré Teaching Hospital and were involved in data collection. Soumaila Sanogo and Abdoulaye Sissoko contributed to data collection and critically reviewed the manuscript. Ibrahima Téguété, as senior principal investigator, oversaw the overall study design and implementation and contributed to data analysis and manuscript writing.

## Funding

This research was based on clinical data routinely collected at Gabriel Touré University Hospital. Cervical cancer screening activities conducted between 2016 and 2023 received financial support from Fondation Orange Mali. In addition, Médecins Sans Frontières‐France (MSF France) supported the management of cervical cancer patients during 2020.

## Disclosure

Dr Tounkara affirms that this manuscript is an honest, accurate, and transparent account of the study being reported; that no important aspects of the study have been omitted; and that any discrepancies from the study as planned have been explained. All authors have read and approved the final version of the manuscript. The funding organizations had no role in the design of the study; the collection, analysis, or interpretation of data; the writing of the manuscript; or the decision to submit the manuscript for publication.

## Conflicts of Interest

The authors declare no conflicts of interest.

## Data Availability

The data that support the findings of this study are available upon request from the corresponding author. The data are not publicly available due to privacy or ethical restrictions.
